# Duplexed direct RNA sequencing protocol using polyadenylation and polyuridylation

**DOI:** 10.1128/mra.01041-24

**Published:** 2025-01-27

**Authors:** Kaylee J. Watson, Robin E. Bromley, Julie C. Dunning Hotopp

**Affiliations:** 1 Institute for Genome Sciences, University of Maryland School of Medicine, Baltimore, Maryland, USA; 2 Department of Microbiology and Immunology, University of Maryland School of Medicine, Baltimore, Maryland, USA; 3 Greenebaum Cancer Center, University of Maryland School of Medicine, Baltimore, Maryland, USA; DOE Joint Genome Institute, Berkeley, California, USA

## Abstract

Oxford Nanopore Technologies provides multiplexing options for DNA and cDNA sequencing, but not for direct RNA sequencing. Here we describe a duplexing approach and validate it by simultaneously sequencing the *Saccharomyces cerevisiae* rRNA from wild type and knockout that have differential rRNA modifications, successfully demultiplexing the data using bioinformatics approaches.

## ANNOUNCEMENT

**Fig 1:**
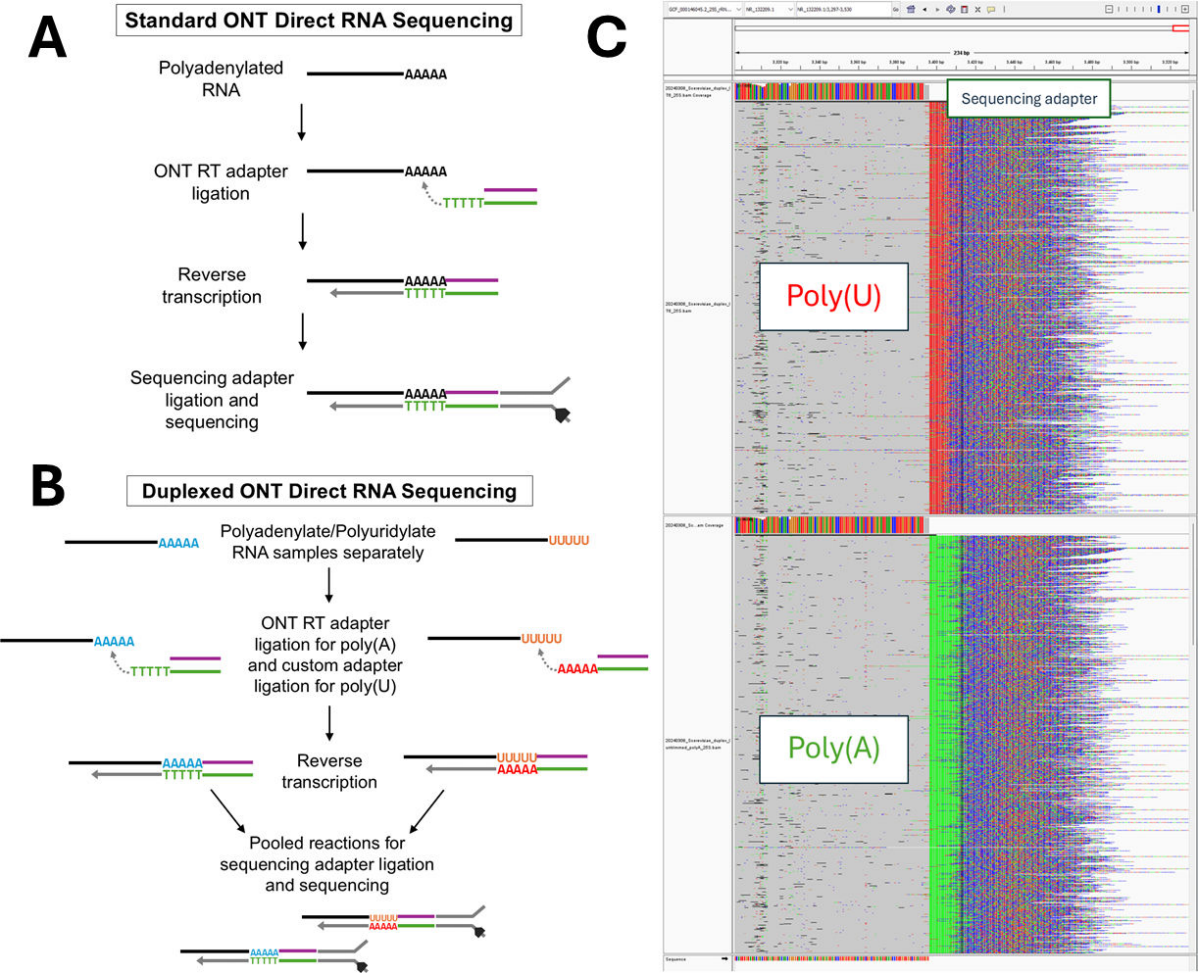
Duplexed ONT direct RNA sequencing. A schematic of the standard ONT direct RNA sequencing method (panel A) is compared to the duplexing method (panel B). An IGV v2.11.2 screenshot (panel C) illustrates that polyuridylated RNA (top with polyuridylation in red) can be differentiated from polyadenylated RNA (bottom with polyadenylation in green).

Oxford Nanopore Technologies (ONT) direct RNA sequencing uses poly(dT) adapters during library preparation to enrich for polyadenylated RNA in a single sample (Fig. 1A). Multiplexed ONT sequencing using commercially available kits is not available for direct RNA sequencing and is currently limited to DNA or cDNA. However, simultaneously analyzing two direct RNA sequencing samples on the same flow cell would be best practice in comparative epitranscriptomics studies, to eliminate any potential biases from using two different flow cells. A protocol for direct RNA sequencing is available that uses custom barcode adapters for multiplexing, but it was developed using the older ONT SQK-RNA002 and SQK-RNA001 chemistries (1). Here, we describe a similar method that facilitates duplex direct RNA sequencing on a single flow cell with the SQK-RNA002 and SQK-RNA004 kits (Fig. 1B), providing access to the raw data generated in this way.

*S. cerevisiae* BY4741 parental and YNL022C RCM1 knockout strains were acquired from Horizon Discovery (cat. no. YSC1048 and YSC6273-201936786, respectively) in August 2022 and subcultured twice. A single colony from 48 to 72 h yeast extract peptone dextrose (YPD) plates grown at 30°C was selected and grown at 30°C with shaking in YPD to OD_600_ = 0.72 for BY4741 and OD_600_ = 0.52 for YNL022C, amending YPD with 200 μg/mL G418 (Enzo Life Sciences) for YNL022C, as recommended in reference 2. Total RNA was isolated using the Zymo YeaStar RNA kit (Zymo Research) according to the manufacturer’s protocol and frozen at −80°C. RNA was quantified on a Qubit fluorometer using the Qubit RNA High Sensitivity kit (QIAGEN).

For BY4741, 1.5 µg (SQK-RNA002) and 1.9 µg (SQK-RNA004) total RNA was polyadenylated using the poly(A) polymerase (New England Biolabs) at 37°C, and for YNL022C, 1.3 µg (SQK-RNA002) and 2.7 µg (SQK-RNA004) total RNA was polyuridylated using the poly(U) polymerase (New England Biolabs) at 37°C, according to the reaction volumes and times in Table 1. Polyadenylated RNA had adapter ligation according to the standard protocol and Table 1, while for polyuridylated RNA a custom adapter containing annealed OligoA (5′-GAGGCGAGCGGTCAATTTTCCTAAGAGCAAGAAGAAGCCAAAAAAAAAA-3′) and OligoB (5′-/5Phos/GGCTTCTTCTTGCTCTTAGGTAGTAGGTTC-3′) (Integrated DNA Technologies) was used in place of the RTA reagent at 1.4 µM concentration. Each reaction was separately reverse transcribed according to the SQK-RNA002/SQK-RNA004 protocols. Since 20 µL from the reverse transcription step is required for sequencing adapter ligation, 10 µL from each reaction was pooled for this step. Direct RNA sequencing was performed on an ONT MinION using an R9 flow cell for SQK-RNA002 and an RNA flow cell for SQK-RNA004 as detailed in Table 1.

**TABLE 1:** Experimental conditions and sequencing results

	SQK-RNA002	SQK-RNA004
Total RNA input for polyA (BY4741)	1.5 µg	1.9 µg
Total RNA input for poly U (YNL022C)	1.3 µg	2.7 µg
PolyA reaction time (BY4741)	30 s	2 min
PolyU reaction time (YNL022C)	2 min	5 min
Library preparation input for polyA (BY4741)	186 ng	177 ng
Library preparation input for polyU (YNL022C)	308 ng	390 ng
Minimum read length filter	None	200 bases
Input RNA amount for sequencing	15 ng	69.36 ng
Total reads sequenced	785,658	5,994,458
Sequenced N50	391 bp	1,177 bp
Mapped reads	363,382	5,077,614
Mapped read percentage	46.25%	84.71%
Supplementary reads	536	82,109
Mapped N50	512 bp	1,295 bp
Max mapped read length	5,247 bp	9,900 bp
rRNA reads	321,984	4,684,946
Percent rRNA (of mapped reads)	88.61%	92.27%
PolyA rRNA reads (BY4741)	247,877	3,002,725
PolyU rRNA reads (YNL022C)	1,102	37,378

FASTQ files were basecalled with Dorado v0.5.1 using the --emit-fastq --no-trim options (3). Alignment was performed using minimap2 v2.24-r1122 (4) with the *S. cerevisiae* S288C R64 reference (5). Default parameters were used except where otherwise noted. Most reads mapped to rRNA (88-92%) (Table 1); position C2278 of the 25S rRNA is known to be methylated by RCM1 in *S. cerevisiae* (6). Using scripts available at https://github.com/kayleewatson/Duplexed-Direct-RNA-Sequencing and SeqKit v0.7.2 (7), the first 45 bases of soft-clipped regions were extracted from the bam files, as this region is expected to contain the poly(A) or poly(U) sequence, represented by a stretch of adenines or thymines. The basecalling process reports uracil as thymine, so poly(T) areas are representative of polyuridylation. EMBOSS fuzznuc v6.6.0.0 -pattern “A(6)” and -pattern “T(6)” was used (8) to search the 45-base regions for stretches of adenines and thymines, then corresponding sequence IDs were used to generate two separate bam files for polyA and polyU reads that can be visualized with IGV v2.11.2 (Fig. 1C) (9). Alignment and demultiplexing statistics are shown in Table 1.

While we describe the use of this method for duplexing yeast RNA, it would be broadly applicable to any experiments where polyadenylation and polyuridylation can be implemented to distinguish between RNA samples.

## Data Availability

All sequencing data have been deposited in the NCBI Sequence Read Archive database (https://www.ncbi.nlm.nih.gov/sra) and assigned the BioProject identifier PRJNA1150648. RNA002 data can be found under accessions SRR30335018 (FAST5) and SRR30335019 (FASTQ), and RNA004 data under accessions SRR30335016 (FAST5) and SRR30335017 (FASTQ). To upload the raw FAST5 data, FAST5 files were basecalled with Guppy v4.2.2, which allows for FAST5 output containing basecalls. Associated FASTQ files were basecalled with Dorado v0.5.1. All commands and scripts for data processing and demultiplexing are available on GitHub: https://github.com/kayleewatson/Duplexed-Direct-RNA-Sequencing (DOI: 10.5281/zenodo.13830111).
